# Negativity in delayed affective recall is related to the borderline personality trait

**DOI:** 10.1038/s41598-022-07358-2

**Published:** 2022-03-03

**Authors:** Aniko Maraz, Tamás Nagy, Matthias Ziegler

**Affiliations:** 1 Humboldt-Universität zu Berlin, Institute of Psychology, Rudower Chaussee 18, 12489 Berlin, Germany; 2 Institute of Psychology, Eötvös Loránd University, Izabella u. 46, Budapest, 1064 Hungary

**Keywords:** Psychology, Human behaviour

## Abstract

The present study assessed selected factors that contribute to the recollection of emotional memories over time. Participants with high-trait borderline personality disorder (BPD) watched a randomly selected positive, negative, or neutral character in a video clip (stimulus) and were asked to recall the content immediately, then 2, 4, and 6 days later. In the final sample (*N* = 558, average age: 33 years, 65% female), general impression had the strongest effect on recall after accounting for the effect of current mood, extremity of the responses, and level of BPD, regardless of stimulus valence. The level of BPD had an effect only when negative evaluative wording (e.g., “guilty”) was used. In conclusion, people with high-trait BPD tend to remember negative stimuli more negatively over time (unlike neutral or positive stimuli), and this effect is mostly related to general impression.

## Introduction

Affective memories prompt a dynamic interaction with the environment and thus influence behaviour. Without adequate emotion regulation, emotion-driven behaviour may lead to severe and lasting consequences. Disrupted emotion regulation is therefore listed as one of the key symptoms of several personality disorders, including borderline personality disorder (BPD)^1^. Patients with BPD experience significantly more intense emotions, greater tension, and more volatile moods than healthy controls^2^. In BPD, one possible source of uncontrolled emotional behaviour is the biased recall of emotional memories. The present study aimed to assess selected factors that potentially contribute to the delayed recollection of emotional impression, given that altered cognitive control processes may be an important factor in emotional behaviour and, in severe cases, suicidal vulnerability^3^.

### Factors influencing recall

There are several factors that potentially influence memory recall, such as mood or cognitive tendencies towards negativity or extremity, and there is evidence that BPD patients may be more affected by these biases than healthy people.

One of the first studies on memory by Nisbett and Wilson^4^ concluded that the global, general impression of a person (e.g., a warm vs. a cold college professor) includes an altered unconscious evaluation of that person’s unrelated attributes (i.e., the attractiveness of their accent), which the authors referred to as the “halo effect”. A more general interpretation of this effect is when an individual with one or several positive (or negative) qualities is assumed to have other positive (or negative) qualities. This has been confirmed in many other studies^5,6^. Negative general impressions of others are particularly pronounced in social interactions in the case of BPD^7^.

Since memories are better recalled when people are in the same affective state^8^, cognitive processes should not be examined as if they were independent of emotional states. However, there is no evidence that current affective state influences learning and memory in BPD patients any differently than in healthy control subjects^9^. In a study by Ebner-Priemer and his colleagues^10^, momentary ratings of current mood were compared to retrospective mood ratings over the same (24-h) period. Within the 24-h period, BPD patients tended to overestimate the intensity of negative mood and underestimate the intensity of positive mood (compared to healthy controls), indicating a valence-dependent recall bias in favour of negativity compared to the non-BPD controls.

The favouring of negativity may, however, be a general cognitive tendency in BPD, rather than being related to mood. For example, the finding that patients with BPD rate momentarily unpleasant emotions with greater intensity and accuracy than pleasant ones has also been confirmed by other studies^1,11,12^, although this phenomenon appears not to be limited to BPD but rather to be a general bias in memory recall^13^. In addition, the tendency towards negativity in BPD has been confirmed by studies using interpersonal stimuli. For example, patients with high-trait BPD showed greater accuracy compared to the low-trait BPD group in decoding negative mental states from photographs of the eye region of various faces, although no significant difference was found between the two groups when neutral or positive stimuli were displayed^11^. Several studies found a bias towards the negative evaluation of others in BPD when patients were asked to judge individuals in film clips^14,15^, while BPD patients also judged their partners more negatively than healthy individuals following a manipulated interaction^16^. Winter and her colleagues^17^ found that BPD patients evaluated negatively and neutrally valenced social events as more negative than healthy controls. However, this effect may not be generalisable to real-world situations^18^.

Patients with BPD are more likely to see the world as all good or all bad—that is, to report more extreme evaluations than healthy individuals. This is known as dichotomous thinking (or splitting)^19^. More specifically, patients with BPD are more likely than healthy controls to give extreme evaluations *at item level*, even if the overall concept is not evaluated as extremely good or bad^15,20^. This is particularly problematic, given that dichotomous thinking is believed to underlie many BPD trait, including affective instability^21^.

Recall bias depends on the temporal distance between the stimulus and the recall. Ebner-Priemer and his colleagues^10^ defined recall bias as the difference between multiple momentary mood ratings (assessed every 10–20 min during waking hours) and a single, retrospective rating of the same 24-h period. They found that BPD patients are likely to recall events in a more negative way, while healthy controls have a more positive recollection. We found no other studies that explored mid-term (1–7 day) recall bias specifically in BPD patients, or in connection with the BPD trait. Studies that focused on immediate recall found evidence of more extreme evaluations among BPD patients (dichotomous thinking)^11,12^ and a tendency towards negative evaluations^14,22^. One study, which explored mid-term (1–7 day) recall bias in healthy participants, found that people remembered positive music better than emotionally neutral music 1 week after presentation, while arousal ratings did not appear to be predictive of recognition^23^.

Although, in the context of interactions, people commonly evaluate others before they act, this is something that has rarely been studied, and the results are inconclusive. When Barnow and his colleagues^14^ asked the participants in their study to evaluate people in neutral film clips, they identified an “aggressivistic evaluation bias”. On the other hand, Veen and Arntz^15^ found trait-level extremity but not dichotomous thinking in the case of BPD-specific film clips, but not in the case of non-specific film clips. Using similar stimuli in a phone call experiment, the same research group found support for dichotomous thinking but not for negativity^16^, whereas manipulated real-life interactions supported the influence of undesirable self-related feedback, and thus of negativity^18^. The question thus remains as to which process influences recall in persons with high-trait BPD, and its relevance to the stimulus.

### Aims and hypotheses

The present study aimed to explore factors that potentially influence emotional, delayed recall, and to investigate the effect of BPD.

The study was pre-registered via the following link: https://osf.io/5249u. The pre-registered hypotheses were the following:

#### H1

Current mood is a stronger determinant of the evaluation of a person’s character over time than extremity, the general impression of the character, or the level of the borderline personality trait (*mood congruency*).

#### H2

The general impression of a person’s character is a stronger determinant of the emotional memory of a video clip over time than extremity or the level of the borderline personality trait (*halo effect*).

#### H3

Extremity is a stronger determinant of valence over time than mood, the general positive impression of a person’s character, or the level of the borderline personality trait (*dichotomous thinking*).

#### H4

The general impression of a person’s character becomes more negative over time, which is positively associated with the level of the borderline personality trait (*negative memory bias*).

#### H5

Valence will turn negative regardless of the original subjective valence of the video clip (*trait-level negativity bias*).

#### H6

The same variables influence the recalled valence of the video clip to the same extent at any point in time.

#### H7

The above effects are valid regardless of video valence.

## Methods

### Data collection procedure

Data were collected via Amazon Mechanical Turk. Adult Americans were invited to take part in the screening and were paid $0.83 or $0.40 to fill out a short screening questionnaire. Inclusion criteria for taking part in the main study were (1) age above 18 years; (2) residence in the USA; (3) McLean Screening Instrument for BPD score > 5 (at least 6 affirmative answers out of a possible 10); and (4) passing the attention check item (“How many full weeks are there in 1 year?”). Only those who met all four screening criteria could participate in the main study. The main study consisted of four waves, approximately 2 days apart, and participants were sent email notifications asking them to fill out the questionnaires. Each participant who completed all four waves received $10 in the form of an amazon.com gift voucher. Data collection is described in detail elsewhere^24^.

### Instruments

Demographic information was collected regarding gender, education, socioeconomic status, and relationship, work, and study status. Current medication and past history of diagnosed BPD were also assessed.

**Figure 1:**
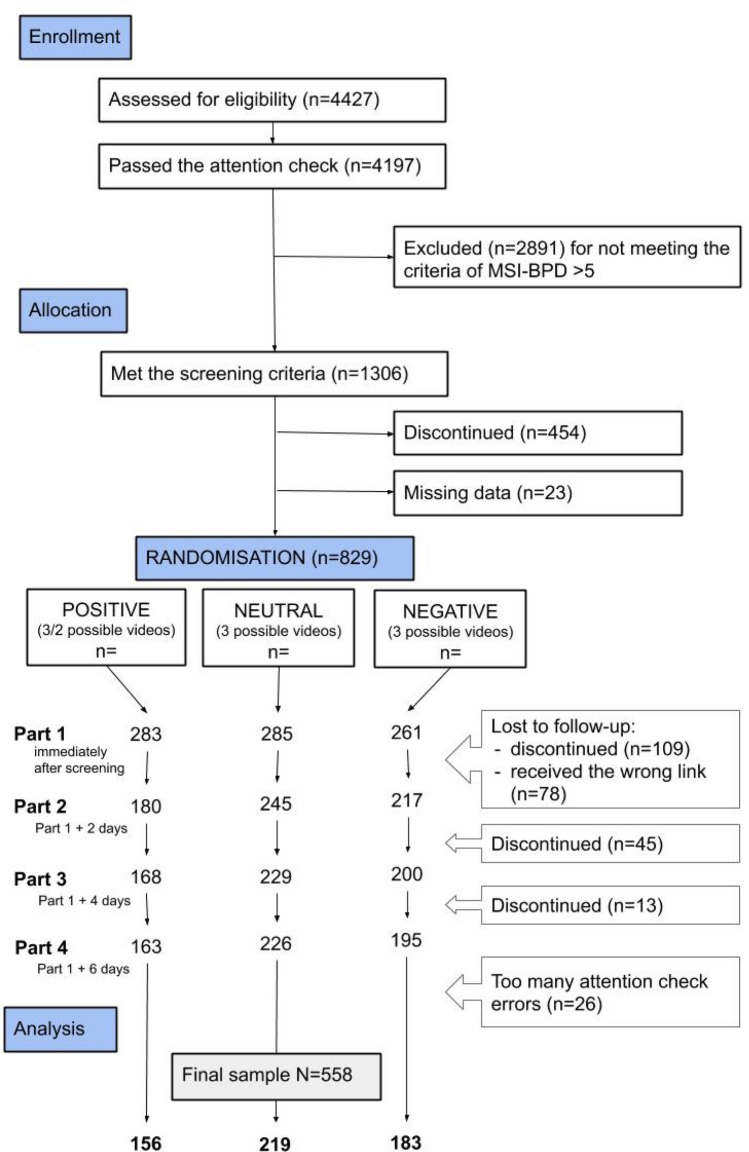
Participant flow chart. *Note*: The figure shows the study design and the number of participants at each stage of the study. In Part 1, participants’ mood was assessed. Participants watched a video clip (which could be positive, neutral, or negative, selected randomly) and filled out questionnaires regarding their general impression and more detailed impression of the character (PANAS). Finally, BPD status was assessed. Only mood, general impression, and detailed impression were assessed during Parts 2, 3, and 4, which took place at intervals of 2 days.

The McLean Screening Instrument for Borderline Personality Disorder (MSI-BPD) was used to screen for BPD on entry (see Fig. 1). The instrument is based on the DSM-IV criteria for BPD and is suitable for use as an index of the severity of BPD symptoms^25^. The 10 items are rated on a dichotomous yes or no scale, with > 6 affirmative answers indicating the probable presence of BPD. The MSI-BPD has acceptable sensitivity (81%) and specificity (85%) when applying the BPD module of the Diagnostic Interview for DSM-IV Personality Disorder^26^ as an external criterion. In the present study, the cut-off was lowered to > 5 affirmative answers, given that sensitivity is better (90%) at this threshold.

The Minnesota BPD scale (MBPD)^27^ was also administered at a later stage in the survey to assess BPD using another independent instrument. Based on an omnibus measure of normal personality (the Multidimensional Personality Questionnaire), the MBPD was validated on two different community samples (undergraduates *N* = 288; young adult twins *N* = 1,132). Evidence for test criterion validity was obtained by testing against the clinical diagnosis of BPD (urban drug users *N* = 146), and cross-validated among prison inmates (*N* = 466). The instrument consists of 21 items structured according to 4 factors that are scored from 1 to 5, and it has good internal reliability in the current sample (Cronbach’s alpha = 0.8). The correlation between the two BPD measures (MSI-BPD and MBPD) was 0.36.

Mood was assessed using the Center for Epidemiologic Studies Depression Scale (CESD)^28^. The 10 items in the scale screen for depressive mood, although the scale is not suitable for diagnostic purposes. Items (e.g., “I felt that everything I did was an effort”) were rated according to past-week frequency on a scale from 1 (“None of the time”) to 5 (“Most of the time”). The CESD score yielded a good reliability estimate (Cronbach’s alpha = 0.85).

Wording, or the positivity or negativity of the instruction, was assessed using the Positive and Negative Affect Scale (PANAS)^29^. The PANAS contains 20 adjectives, 10 of which are negative (e.g., “irritable”, “hostile”) and 10 positive (e.g., “excited”, “strong”). Participants were asked to rate the characters in the video clips according to each scale—that is, “How irritable do you think the woman was in this clip?” or “How determined do you think the woman was in this clip?” Ratings were possible between 1 (“Very slightly or not at all”) and 7 (“Extremely”). Both scores—the score for the positive subscale (positive evaluation, e.g., “excited” or “jittery”) and the score for the negative subscale (negative evaluation, e.g., “guilty” or “scared”)—had excellent reliability estimates in the current sample (Cronbach’s alphas were 0.88 and 0.89 respectively).

Extremity was defined as extreme choices on the short, 20-item version of the International Personality Item Pool (Mini-IPIP)^30^, a questionnaire based on the big five personality traits. Items were rated on a scale from 1 to 5. When calculating extremity, every rating of 1 and 5 was scored as 2; every rating of 2 and 4 was scored as 1; and every rating of 3 was scored as 0. The final extremity scale therefore ranged between 0 and 40.

The test video clips were taken from Schaefer et al.^31^. The clips were selected to (1) represent a clearly neutral, positive, or negative interaction (as assessed by the authors); and (2) feature a clear principal character. Clips within valence groups were as heterogeneous as possible in terms of arousal, as reported in the paper^31^. A trial clip (same for every participant) preceeded the test video clip to practice and exclude technical issues.

All items within the same instrument were displayed to participants in random order to minimise the effect of order of presentation. A summary of the instruments administered in the present study is provided in Table 1.

**Table 1:** Measurements at each stage of the study.

	Screening	Part 1	Part 2	Part 3	Part 4
Consent	x				
Demographics	x	x			
Mood (CESD)		x	x	x	x
Trial video		x			
General positive impression of the character (“How much did you like the character of the woman [pictured above] in this clip?”)		x	x	x	x
Qualitative (“Please tell us your impression of the character of the woman in this clip.”)		x			
Test video (positive/neutral/negative)		x			
General positive impression of the character (“How much did you like the character of the [woman] (pictured above) in this clip?”)		x	x	x	x
Qualitative assessment (“Please tell us your impression of the character of the [woman] in this clip”)		x	x	x	x
Quantitative assessment (PANAS, with positive and negative evaluative wording scales, e.g., “How irritable do you think the woman was in this clip?”)		x	x	x	x
Borderline personality trait	x_(McLean)_	x_(Minnesota)_			
Life events (past week)		x			x_(since Part 1)_

Part 1 immediately followed screening; Part 2 was offered 2 days thereafter; Part 3 two days after Part 2; and Part 4 two days after Part 3.

### Data cleaning and validation

Several measures were taken to improve data quality, as reported in the earlier publication^24^. Firstly, two attention check items were hidden among the regular items (“Click <extremely> here” and “Do you agree that dogs need water to survive?”), and one item was added to check whether participants had watched the critical (second) video clip (e.g., “How did the woman die in this clip?” Alternative answers: “She slipped and fell off her bike/She was hit by a truck/She was hit by a car”). Secondly, participants were asked to report their residence, gender, and age at screening and again later during the survey. Each time the answers did not match, the participant scored an error. Finally, we measured the time spent on the trial and the test video pages, and participants scored an error when the time spent on the page was less than the length of the video, based on the assumption that they had not watched the video in full. Participants who scored more than 2 errors on the attention check items (out of a possible 15) were excluded from the sample^24^.

### Sample

A total of 4,719 people clicked on the survey website, and 4,427 filled out the screening questionnaire. Of these, 1,306 (29.5%) screened positive on the MSI-BPD and were offered the opportunity to take part in the study.

The interval between the different parts of the study was set to 48 h. When the time expired, participants received a reminder and had another 48 h to respond. Exceeding this deadline led to the termination of the questionnaire (dead end by design). The median time between screening positive and completing Part 1 was 5 min; between Part 1 and Part 2 it was 52 h; between Part 2 and Part 3 it was 53 h; and between Part 3 and Part 4 it was 51 h (meaning that participants typically reacted 3 to 5 h after receiving the email).

Data for Part 1 were provided by 829 participants (63.5%). There was no difference on the BPD screening scale (MSI-BPD) between those who wanted to take part in the study and those who did not (*t* = − 0.029; *df* = 1,005.5; *p* = 0.98). Participants who screened positive were randomised to one of the nine groups. Each participant received two video clips: a test video (the same for every participant), plus a target video that was randomly selected from nine possible clips (with positive, neutral, or negative valence). Unfortunately, Group 2 received the incorrect link to the video (the same link as for Group 1), therefore these data had to be excluded (*N* = 78). Out of the remaining eight groups, totalling 751 participants, 167 participants discontinued the survey despite the reminders. Finally, data for 26 participants had to be excluded due to too many (> 2) errors on the attention check items, leaving 558 participants’ data for the final analysis. Details are shown in Fig. 1.

### Procedure

Eligible participants filled out questionnaires about demographics and mood. They then watched two video clips (see below) before filling out the rest of the questionnaires. All the video clips were selected according to the validation study reported by Schaefer et al.^31^. One of the short video clips, taken from the film “A Fish Called Wanda”, was shown to all participants as a technical check (to test video and audio quality). The second clip was chosen randomly from nine possible video clips. In order to be included, the clips had to (1) represent a social interaction; (2) contain a clear principal character; and (3) represent a clearly positive (e.g., tender) or a clearly negative (e.g., angry or fearful) interaction, or a clearly neutral scene (e.g., a woman walking through a market). Three clips were selected within each valence group that were as heterogeneous as possible in terms of intensity (arousal). The clips in the valence groups were similar in terms of the character with whom the participants were asked to identify (e.g., “young woman”)^24^. The selected clips are available on the OSF website (see “Ethics, data availability, and pre-registration” section for the link).

Firstly, the general positive impression of the character (not of the video clip) was assessed: “How much did you like the character of the woman (pictured above) in this clip? Please make sure that you rate the character in this short clip and not the actress or the movie.” Possible ratings ranged between 1 (“Disliked”) and 7 (“Liked”). Secondly, an open-ended question was asked, focusing specifically on the target character in the clip (e.g., “Please tell us your impression of the character of the woman in this clip as you would tell it to a friend. Please make sure that you recall as many details as you can.”). Finally, the participant’s emotional attitude towards the character in the video clip was assessed using the PANAS (described above).

### Data analysis

The survey was created and all the data were collected using formr^32^. The data were analysed and visualised in R^33^ and the psych^34^, ggplot2^35^, Hmisc^36^, lmer^37^, lmerTest^38^, and sjstats^39^ packages. All the data, scripts, and results presented here (along with complementary analyses) are available on the OSF website (see “Ethics, data availability, and pre-registration” section for the link).

To investigate whether the clips were able to elicit the intended emotions, we used pairwise t-tests on positive and negative evaluations of the character (PANAS) and on the general positive impression of the character, with the video valence group as the independent variable.

In order to test hypotheses H1–H5 and H7, the participant’s mood, the general positive impression of the character, extremity, and a negative or positive evaluation of the character (PANAS) were used as outcomes, with valence group, time, and valence group x time interaction and BPD trait as predictors in random intercept linear mixed-effect models.

Multiple linear regression models were used to test H6 (and H7). In total, 24 regressions were conducted with the positive (PANAS Positive) and negative (PANAS Negative) evaluation of the character as dependent variables. Models were run separately for each time point and each video valence group (positive, neutral, and negative). Predictors were BPD trait, participant mood, general positive impression of the character, and extremity. All predictors were entered in the models together. Continuous variables were centred and scaled in all models, and values were adjusted using false discovery rate (FDR) correction.

### Ethics, data availability, and pre-registration

The study is in line with the Declaration of Helsinki and received institutional review board approval from the Humboldt University of Berlin (2018-05-R). Informed consent was obtained from all participants before they joined the study. The present paper, including the hypotheses and data analytical plans, was pre-registered on the Open Science Framework (OSF) website under the following link: https://osf.io/5249u. All materials, other publications containing hypotheses and data analysis scripts, and the complete data are available on the OSF website: https://osf.io/nuqy8/.

## Results

### Descriptive statistics

The average age of the participants was 33 years (*SD* = 9.3); 65% were female, 34% male, and 1% indicated “other” gender. In terms of education, 42% had completed vocational, secondary, or primary school; 45% had an undergraduate degree; and 13% had a graduate degree. Participants reported below-average living circumstances: 35% reported being “among the poorest”; 50% reported being poor or below average; and 4.5% reported having an average income. The majority (87%) were American, and 98% spoke English as their native language. While 57% had a full-time job, 75% were studying. Of the 69% who reported being in a relationship, 52% were married. Around a quarter (27%) reported taking psychotropic medication (e.g., tranquillisers and antidepressants, based on the free-text answers) and 6.1% had received a BPD diagnosis. At the start of the study, 156 participants (28%) were randomly assigned to the positive video valence group, 219 (28%) to the neutral group, and 183 (33%) to the negative group. Most of the variables were normally distributed (see Supplement 1 for details).

### Evaluation of the clips

**Figure 2:**
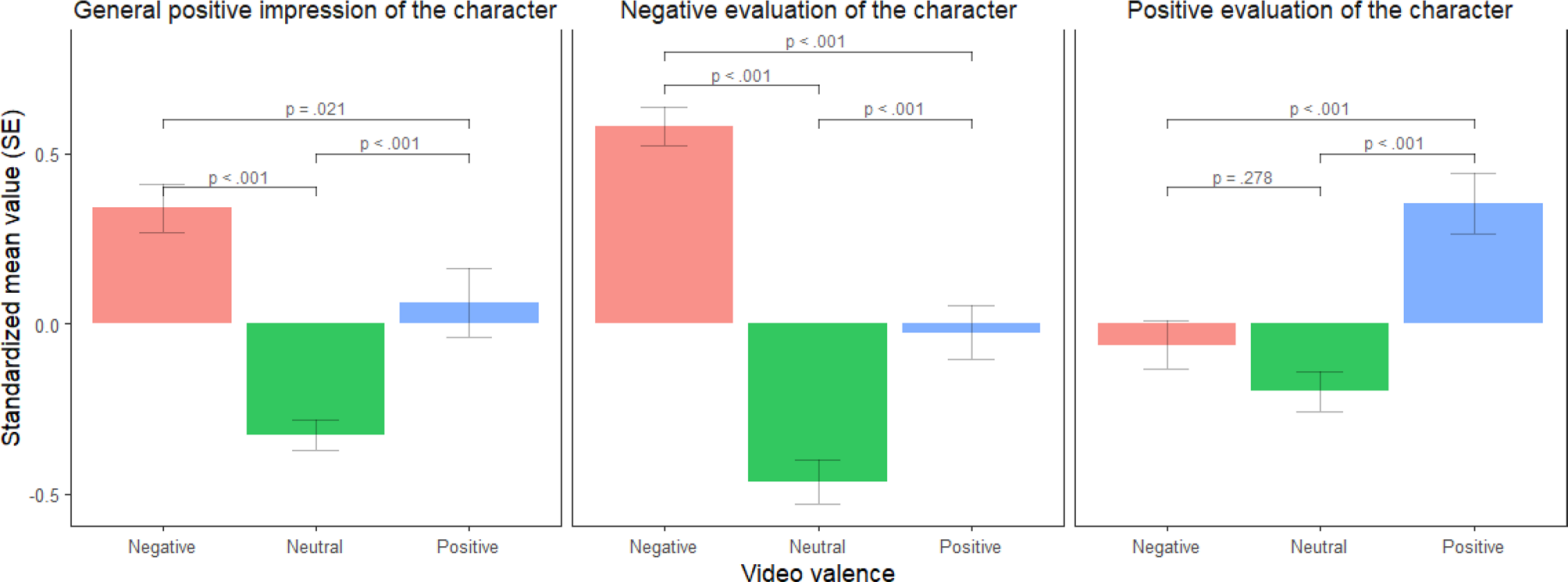
The effect of manipulation by valence group.

Firstly, we checked whether the participants evaluated the valence of the characters in the video clips as we intended. Characters were rated according to our intentions on PANAS negative and positive scales. However, the one-item general impression of the character was the most positive for the negative clips and the least positive for the neutral clips. Details are shown in Fig. 2.

### Determinants of character evaluation (H1, H2 and H3)

As shown in Supplement 2, the positive evaluation of the character differed according to group (video valence had a significant main effect in each model, with an effect size between 0.06 and 0.23, with higher values in the negative evaluation models). It also showed a distinct trajectory over time (valence x time interaction) in the positive evaluation models (small effect sizes between 0.01 and 0.02). The level of BPD trait had a significant main effect in all models with negative character evaluation as the dependent variable (effect sizes between 0.02 and 0.04), but not in models with positive character evaluation.

In terms of the hypotheses, participants’ mood and the general impression of the character had a main effect on both (positive and negative) character evaluations (confirming H1 and H2), whereas the extremity of the responses did not (weakening H3) after accounting for potential overlapping (main and interactional) effects between the variables tested in this study.

### Change in impression over time (H4 and H5)

**Figure 3:**
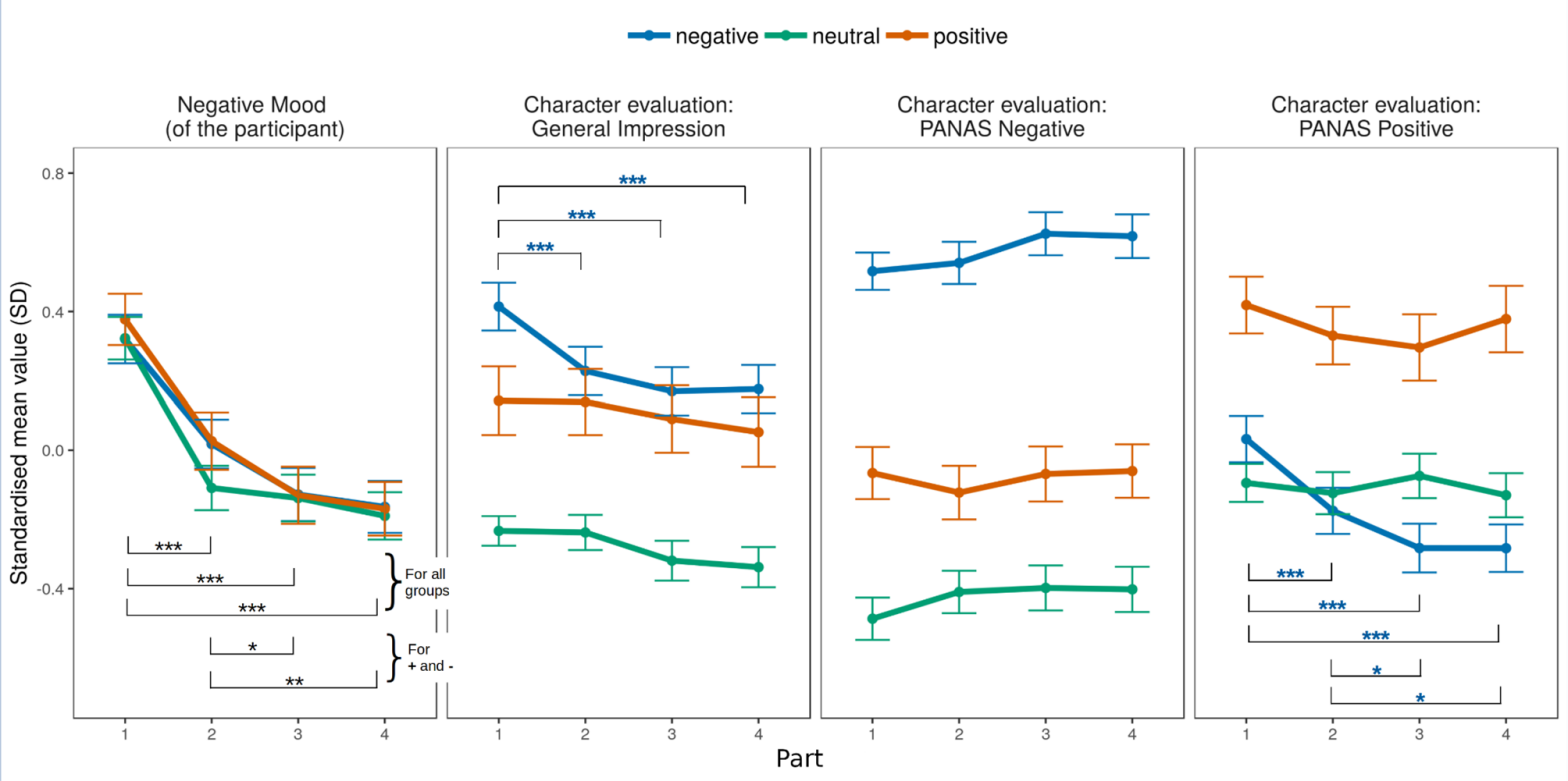
Changes over time in participants’ mood and character evaluations per valence group. *Note*: Significant pairwise tests for the repeated measures within groups are indicated by symbols. Follow-ups (study parts) took place approximately 2 days apart. Error bars represent the standard error of the mean. The general positive impression of the character was rated negative to positive.

The general positive impression of the character changed over time: group *F*(2, 552) = 18.67, *p* < 0.001, η_p_^2^ = 0.05; time *F*(3, 1656) = 12.66, *p* < 0.001, *HF* ε = 0.805, η_p_^2^ = 0.003; group × time *F*(6, 1656) = 2.42, *p* = 0.025, *HF* ε = 0.805, η_p_^2^ = 0.001. However, post hoc tests revealed that only the negative valence group showed a significant change over time in the general impression of the character: this group recalled the character in the video as significantly more negative on each occasion compared to the first assessment (see Fig. 3). When entered in a multilevel regression with general positive impression of the character as dependent, and time and the level of BPD as independent predictors, time had a significant effect, whereas BPD trait had a non-significant effect: *t*(180.98) = 0.592, *p* = 0.555. Thus, H4 was partially confirmed: the general impression of the character became more negative over time, but only within the group that watched the negative video clips, and this was unrelated to the level of the BPD trait.

The evaluation of the characters was investigated using both negative and positive evaluative wording. Negative evaluation was predicted by valence group: *F*(2, 552) = 74.22, *p* < 0.001, η_p_^2^ = 0.18; time: *F*(3, 1656) = 3.25, *p* = 0.021, *HF* ε = 0.881, η_p_^2^ = 0.001; and group x time: *F*(6, 1656) = 1.052, *p* = 0.39, *HF* ε = 0.881, η_p_^2^ < 0.001. Similarly, in the case of positive evaluation, both main effects and the interaction were significant in the model (group: *F*(2, 552) = 17.63, *p* < 0.001, η_p_^2^ = 0.05; time: *F*(3, 1656) = 11.70, *p* < 0.001, *HF* ε = 0.922, η_p_^2^ = 0.003; interaction: *F*(6, 1656) = 6.98, *p* < 0.001, *HF* ε = 0.922, η_p_^2^ = 0.004). The change in the positively worded evaluations (PANAS Positive) over time was unrelated to the level of BPD trait in the negative valence group when entered into a multilevel regression with valence as dependent and time and BPD as independent predictors: *t*(180.72) = 1.511, *p* = 0.133.

**Figure 4:**
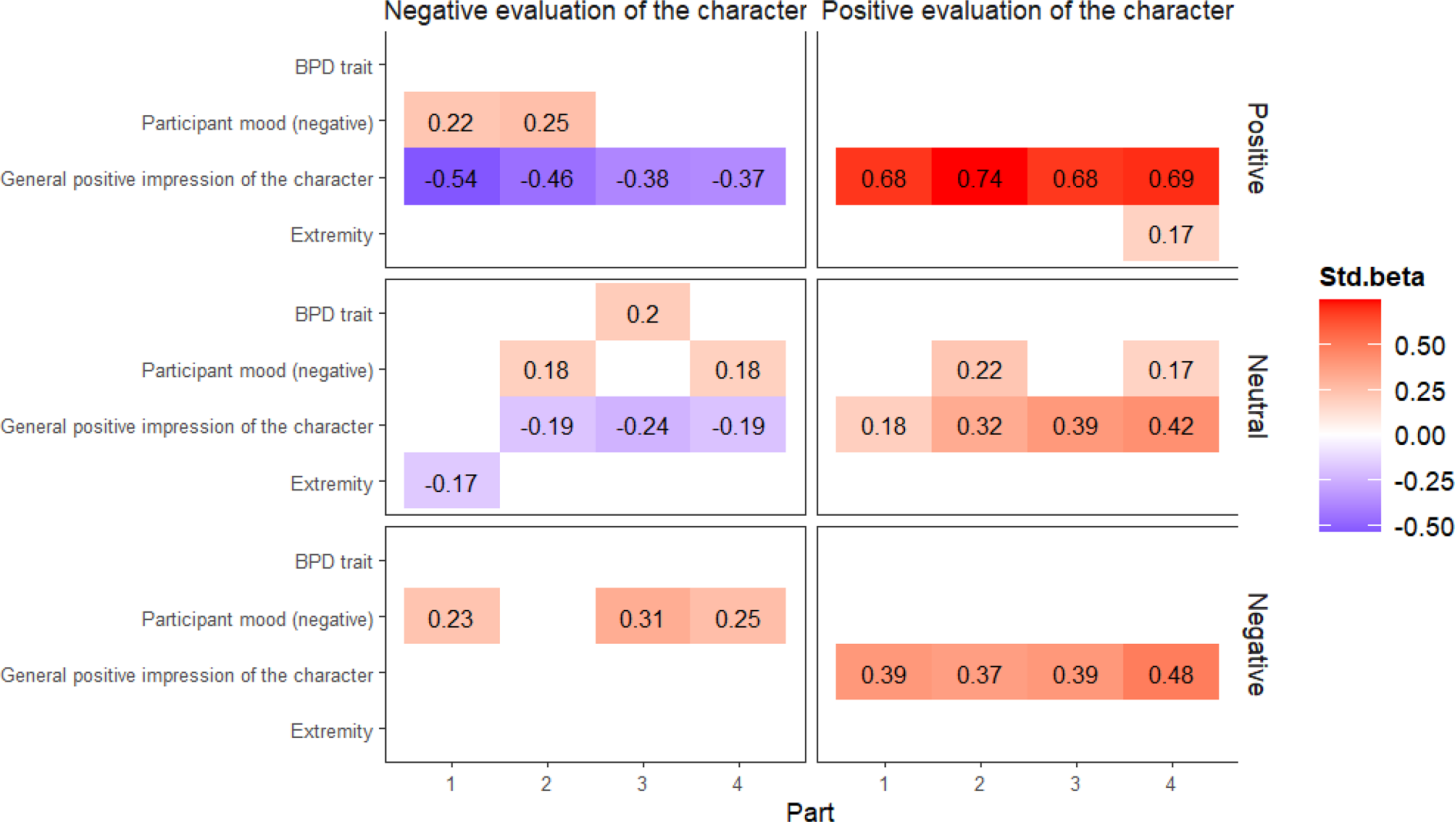
Predictors of character evaluation at each point of assessment by video valence. *Note*: “Part” refers to the time of measurements, which took place at intervals of approximately 2 days. Values are standardised betas from linear regression models. Only the significant predictors are shown; p values were adjusted using FDR correction. In total, 24 separate models were conducted. The outcome variable was either the positive evaluation of the character or the negative evaluation of the character. Models were conducted separately by video valence (positive, neutral, or negative) and by time. We used BPD trait, participants’ negative mood, the general positive impression of the character, and the extremity of the responses as predictors.

Supplement 3 shows changes over time according to BPD severity group. (These were used for visualisation purposes only: the BPD trait was entered as a continuous variable in the analyses.) As shown in Fig. 4, participants with a medium or high level of BPD trait evaluated all the videos (negative, neutral, and positive) more negatively than low-trait BPD participants when assessed using negative wording (character evaluation), but this effect disappeared when using positive character evaluation. Furthermore, participants with high-trait BPD tended to evaluate the positive (and to a lesser extent the neutral) videos more negatively than the other groups.

The data thus partially confirmed H5, since the negative (but not the neutral or positive) videos were evaluated as decreasingly positive over time, although this was found only when positive evaluative wording rather than negative evaluative wording was used. This effect was unrelated to the level of BPD trait, although participants with a high level of BPD trait consistently evaluated characters more negatively over time, regardless of valence.

### Models at each moment of assessment (H6 and H7)

As summarised in Fig. 4, in the positive valence condition the general impression of the character (to a decreasing extent over time) was a significant predictor of attitude (positive or negative PANAS) towards the character at all timepoints, with strong effect sizes. On the other hand, a positive attitude (PANAS) towards the character was mostly predicted by the general impression of the video (and to a far smaller extent by the participant’s mood in Part 4). In the negative valence group, the general impression of the character remained a significant predictor, regardless of the time of assessment, but only for character evaluation using positive wording. Negative wording was predicted by participant’s mood and not by general impression. General impression again played an important role in the recollection of neutral videos: general impression predicted both a positive, and to a lesser extent negative, evaluation of the character. The level of BPD trait influenced the negative evaluation of the character only in Part 3, but had only a small effect.

Regarding larger effects, it therefore appears that similar (although not identical) factors influenced the recollection of the video clips in each part of the study, which partially confirms H6.

The aim of H7 was to explore whether the above differences were related to video valence. As evidenced in the model testing for H1–H3, video valence had a main effect in the prediction models, meaning that the effect of the variables in the model differed according to the valence of the video. Regarding changes over time (H4 and H5), participants in the negative valence group recalled the character in the video significantly more negatively over time compared to the first assessment (see Fig. 3 and “Change in impression over time (H4 and H5)” section). A general positive impression of the character clearly influenced the responses to both the negative and positive evaluation of the character when participants were confronted with a positive stimulus (see the analysis for H6). This effect was apparent, although far weaker, in the case of neutral stimuli. However, only the positive evaluation of the negative stimulus was influenced by the general impression of the character, while the negative character evaluation of the negative stimulus remained unaffected (see Fig. 4).

## Discussion

In the present study we tested several factors that may influence emotional recall over time, paying special attention to changes related to the level of BPD trait. Following four assessments over 1 week, we found that the general positive impression of the character had the strongest effect on recall, and that negative stimuli were more negatively evaluated over time. However, these effects were largely independent of the level of BPD. On the other hand, the level of BPD did influence the evaluations when the recall instruction was negatively worded (e.g., “guilty”), but not when it was positively worded (e.g., “enthusiastic”).

These findings are valid regardless of the video valence (negative, positive, or neutral). We found no evidence of the effect of mood congruency or dichotomous thinking after accounting for all other factors. Different factors contributed to the formation of memory over time, both confirming and weakening existing theories in the field.

We found that mood and the general positive impression of the character, but not the extremity of the responses, influenced negative evaluations of the memory independently from one another. This confirms H1 and H2 but weakens H3. The general positive impression of the character changed only within the negative valence group (and independently of the level of BPD trait), partially confirming H4. Negative (but not neutral or positive) videos were evaluated as decreasingly positive over time, but only when positive evaluative wording was applied to the character (and not in the case of negative wording), and this was unrelated to BPD, which partially confirms H5. On the other hand, general impression had a marked influence on the responses (independently from BPD) when the evaluations were obtained using positive wording for negative videos, while the level of BPD trait had a minor influence on the responses only when the evaluations were obtained using negative wording (H6). This statement is valid regardless of time. The evaluation of H7 is more complex. The general positive impression of the character influenced responses obtained with positive evaluative wording regardless of the valence of the stimulus (positive, neutral, or negative, although the strongest effect was observed with positive videos) and with negative evaluative wording in the case of a positive stimulus, although this effect was far weaker in the case of neutral stimuli and non-existent in the case of negative stimuli (H7).

Recall is an active process that is likely to distort the memory of past experiences. Our findings may help to clarify memory functions in relation to BPD. Firstly, after accounting for overlapping between the variables, the biggest influence on recollection is the general positive impression of the character, known as the halo or anchoring effect, and not mood congruency, negativity bias, dichotomous thinking, or the extremity of the responses. The halo effect is an adaptive coping strategy, since over‐general autobiographical recall may help to protect borderline individuals from parasuicidal acts by helping them to avoid distressing memories^40^. According to our results, this is particularly true when the memory is accessed via positive wording, regardless of whether the initial experience was negative, neutral, or positive (although the effect is strongest in the case of positive stimuli). Recall accessed via negative wording did not fully reflect the true valence of the initial experience. Mixed evaluative wording in past studies (positive–negative visual analogue scales) may therefore have masked this effect^15^. Our findings show that, although those with a higher level of BPD trait tend to overestimate the valence of mood states retrospectively^10^, this does not influence their responses when recalling information. More importantly, we found that this effect is unrelated to the level of BPD trait.

When the memory was accessed via negative evaluative wording, those with a high level of BPD trait were more likely to recall events more negatively than those with low or medium BPD trait levels, especially when the event was negative or neutral. This is in line with previous findings^10,22,41^. However, our data show that this bias is not related to the level of BPD trait after accounting for overlaps with other variables (e.g., the extremity of the responses) but is rather the result of a general cognitive tendency towards sensitisation^42^. It is possible that only patients with high levels of BPD trait are likely to report frequent and intense negative memories, compared to healthy controls^1^. Other evidence suggests that negative (and not positive) information may be more self-relevant to those with BPD, due to their negative self-concept^17^.

The increasing negativity of interpersonal evaluation may be a negative spiral that is probably sustained by the lack of positive general impression. Other authors also report that negative, but not neutral or positive, memories tend to be anchored as a generally negative image that becomes more negative over time. This increases the likelihood of depressive and stress symptomatology^43^. However, we found that this was true only if the memory was accessed via positive wording, while the effect was far weaker in the case of negative wording. This effect may reflect the discrepancy between the desired and the real valence of the memory, and this discrepancy results in the maintenance of the negative spiral^44^.

We also found that participants with a high level of BPD are sensitive to negative wording in the instruction, which influences the responses independently of all other factors. After accounting for other variables, the effect of BPD trait level disappears when positive wording is used in the recall instruction. These findings are probably not due to cognitive performance impairment^45^ but rather to a characteristic related to the level of BPD trait.

Interpretive bias can be effectively trained^46^. For example, positive retrieval training yields a stable positive mood, which also transfers to autobiographical memory^47^. When trained, participants interpret ambiguous information in a way that is congruent with their positive or negative training^48^. This is especially important, because higher variability in affect (negative and positive) predicts alcohol use in patients with BPD^49^, thus the training of interpretive bias is likely to result in the reduction of BPD symptoms.

Our findings are limited to the sample we obtained via MTurk, and future studies should verify whether these findings can be generalised to other samples. By design, we relied on screening and self-reporting, thus clinical studies should be carried out to confirm the results. However, given that we entered the cumulative BPD score rather than the categorisation (i.e., BPD vs. non-BPD), the lack of clinical diagnosis may not have had a major distorting effect on the results. Furthermore, we ignored general cognitive skills (e.g., intelligence) and relied instead on data cleaning, although earlier research has shown that BPD patients do not differ from healthy controls in terms of their ability to memorise emotional information^9^. Finally, it is possible that the above markers are not limited to BPD but have cross-diagnostic specificity. Previous studies have reported mixed findings: Houben^50^, for example, found that emotional switching was not specific to BPD compared to bulimia, PTSD, and depression, while others found that emotional intensity was typical of BPD^51^. In addition, the single-item measure of general impression has a lower reliability than the multi-item measure of positive and negative character evaluation (PANAS), which may explain why general impression did not have the pattern that we expected based on the previously determined valence of the videos. Further caution is needed, given the high proportion of participants taking psychotropic medication (27%), although this does not reflect the prevalence of prescription medicine use among the U.S. population (45.7% between 2015 and 2018, source: https://www.cdc.gov/nchs/data/hus/2019/039-508.pdf).

To conclude, we identified at least three important findings. Firstly, the general positive impression of the character had a marked influence on recall (independently of BPD), regardless of the stimulus valence (positive, neutral, or, to a lesser extent, negative). This effect was particularly strong when the evaluations used positive wording. The halo effect was thus detectable when the memory was accessed via positive evaluative wording, although this finding was unrelated to the level of BPD trait. Secondly, trait-level negativity bias was detectable only in the negative stimulus/positive evaluative wording condition and was unrelated to the level of BPD trait. Thirdly, the level of BPD trait had an effect on the responses only in the case of negative evaluative wording (but not with positive), and this effect was detectable even after controlling for the extremity of the responses (dichotomous thinking), general impression (halo effect), participant’s mood (mood congruency), and trait-level negativity. Future studies should therefore account for the fact that those with high-trait BPD are more sensitive to negative wording in the instruction, regardless of stimulus valence.

## Supplementary Information

Supplementary Information 1.

Supplementary Information 2.

Supplementary Information 3.
