# Supplementary material for: Negativity in delayed affective recall is related to the borderline personality trait
Source: Sci Rep. 2022 Mar 3;12:3505. doi: 10.1038/s41598-022-07358-2 (PMC8894358; doi:10.1038/s41598-022-07358-2)
Supplement: Supplementary file 1 — Supplementary Information 1. [file 41598_2022_7358_MOESM1_ESM.docx]

**Supplementum 1**

**Descriptive statistics and distributions of key variables**

| **Variable** | **Theoretical** | | **Empirical** | | | | **Distribution** |
| --- | --- | --- | --- | --- | --- | --- | --- |
|  | **Min** | **Max** | **Mean** | **SD** | **Min** | **Max** |  |
| Extremity | 0 | 40 | 23.91 | 6.19 | 5 | 40 | ▁▂▆▇▇▃▁ |
| BPD trait | 21 | 84 | 55.35 | 7.68 | 30 | 76 | ▁▂▃▇▆▃▁ |
| Participant mood (negative) | 10 | 50 | 28.61 | 6.56 | 10 | 47 | ▁▃▅▇▅▃▁ |
| Positive evaluation of the character | 10 | 70 | 38.48 | 13.65 | 10 | 70 | ▂▅▇▇▆▃▂ |
| Negative evaluation of the character | 10 | 70 | 34.25 | 14.53 | 10 | 70 | ▇▆▅▇▆▃▁ |
| General positive impression of the character | 1 | 7 | 4.71 | 1.70 | 1 | 7 | ▂▂▃▇▆▅▆ |

*Note: Scale description for the variables used in this study.*
