# Supplementary material for: Negativity in delayed affective recall is related to the borderline personality trait
Source: Sci Rep. 2022 Mar 3;12:3505. doi: 10.1038/s41598-022-07358-2 (PMC8894358; doi:10.1038/s41598-022-07358-2)
Supplement: Supplementary file 2 — Supplementary Information 2. [file 41598_2022_7358_MOESM2_ESM.docx]

**Supplementum 2**

**Determinants of the character evaluation over time (H1, H2 and H3)**

|  | **Positive evaluation of the character** | | | | **Negative evaluation of the character** | | | |
| --- | --- | --- | --- | --- | --- | --- | --- | --- |
| **Parameter** | **F** | **df** | **p** | **η^2^_p_** | **F** | **df** | **p** | **η^2^_p_** |
| **H1: Model for participant mood** | | | | | | | | |
| Video valence | 17.09 | 2, 552 | **<0.001** | 0.058 | 77.24 | 2, 552 | **<0.001** | 0.218 |
| Time | 12.27 | 1, 1668 | **0.001** | 0.007 | 21.86 | 1, 1668 | **<0.001** | 0.013 |
| Participant mood (negative) | 14.66 | 1, 552 | **<0.001** | 0.007 | 38.29 | 1, 552 | **<0.001** | 0.018 |
| BPD trait | 0.08 | 1, 552 | 0.776 | <0.001 | 12.07 | 1, 552 | **0.001** | 0.020 |
| Video valence × Time | 12.21 | 2, 1668 | **<0.001** | 0.014 | 1.35 | 2, 1668 | 0.381 | 0.002 |
| Video valence × Participant mood (negative) | 1.16 | 2, 552 | 0.415 | 0.001 | 2.78 | 2, 552 | 0.114 | 0.003 |
| Time × Participant mood (negative) | 1.43 | 1, 1668 | 0.342 | 0.001 | 0.11 | 1, 1668 | 0.790 | <0.001 |
| Video valence × Time × Participant mood (negative) | 1.33 | 2, 1668 | 0.374 | 0.002 | 0.92 | 2, 1668 | 0.474 | 0.001 |
| **H2: Model for General positive impression** | | | | | | | | |
| Video valence | 22.76 | 2, 552 | **<0.001** | 0.079 | 86.81 | 2, 552 | **<0.001** | 0.236 |
| Time | 9.87 | 1, 1668 | **0.004** | 0.006 | 2.22 | 1, 1668 | 0.211 | 0.001 |
| General impression of the character | 191.22 | 1, 552 | **<0.001** | 0.082 | 45.46 | 1, 552 | **<0.001** | 0.021 |
| BPD trait | 1.52 | 1, 552 | 0.338 | 0.003 | 21.95 | 1, 552 | **<0.001** | 0.038 |
| Video valence × Time | 10.52 | 2, 1668 | **<0.001** | 0.013 | 1.09 | 2, 1668 | 0.457 | 0.001 |
| Video valence × General impression of the character | 11.47 | 2, 552 | **<0.001** | 0.011 | 9.29 | 2, 552 | **<0.001** | 0.009 |
| Time × General impression of the character | 3.23 | 1, 1668 | 0.125 | 0.002 | 4.96 | 1, 1668 | 0.054 | 0.003 |
| Video valence × Time × General impression of the character | 0.77 | 2, 1668 | 0.496 | 0.001 | 2.81 | 2, 1668 | 0.114 | 0.003 |
| **H3: Model for Extremity** | | | | | | | | |
| Video valence | 16.10 | 2, 552 | **<0.001** | 0.055 | 75.18 | 2, 552 | **<0.001** | 0.214 |
| Time | 26.91 | 1, 1668 | **<0.001** | 0.016 | 8.61 | 1, 1668 | **0.008** | 0.005 |
| Extremity | 0.73 | 1, 552 | 0.451 | 0.001 | 2.71 | 1, 552 | 0.173 | 0.005 |
| BPD trait | 0.77 | 1, 552 | 0.451 | 0.001 | 22.95 | 1, 552 | **<0.001** | 0.040 |
| Video valence × Time | 13.88 | 2, 1668 | **<0.001** | 0.016 | 1.04 | 2, 1668 | 0.457 | 0.001 |
| Video valence × Extremity | 1.13 | 2, 552 | 0.415 | 0.004 | 2.22 | 2, 552 | 0.179 | 0.008 |
| Time × Extremity | 4.26 | 1, 1668 | 0.072 | 0.003 | 0.01 | 1, 1668 | 0.939 | <0.001 |
| Video valence × Time × Extremity | 2.06 | 2, 1668 | 0.209 | 0.002 | 0.36 | 2, 1668 | 0.790 | <0.001 |

Notes: Omnibus tests for six separate linear mixed-effect models. P values were adjusted using FDR correction.
