# Supplementary material for: Negativity in delayed affective recall is related to the borderline personality trait
Source: Sci Rep. 2022 Mar 3;12:3505. doi: 10.1038/s41598-022-07358-2 (PMC8894358; doi:10.1038/s41598-022-07358-2)
Supplement: Supplementary file 3 — Supplementary Information 3. [file 41598_2022_7358_MOESM3_ESM.docx]

**Supplement 3**

**Changes in the evaluation of the videos and the characters over time for different levels of BPD trait**


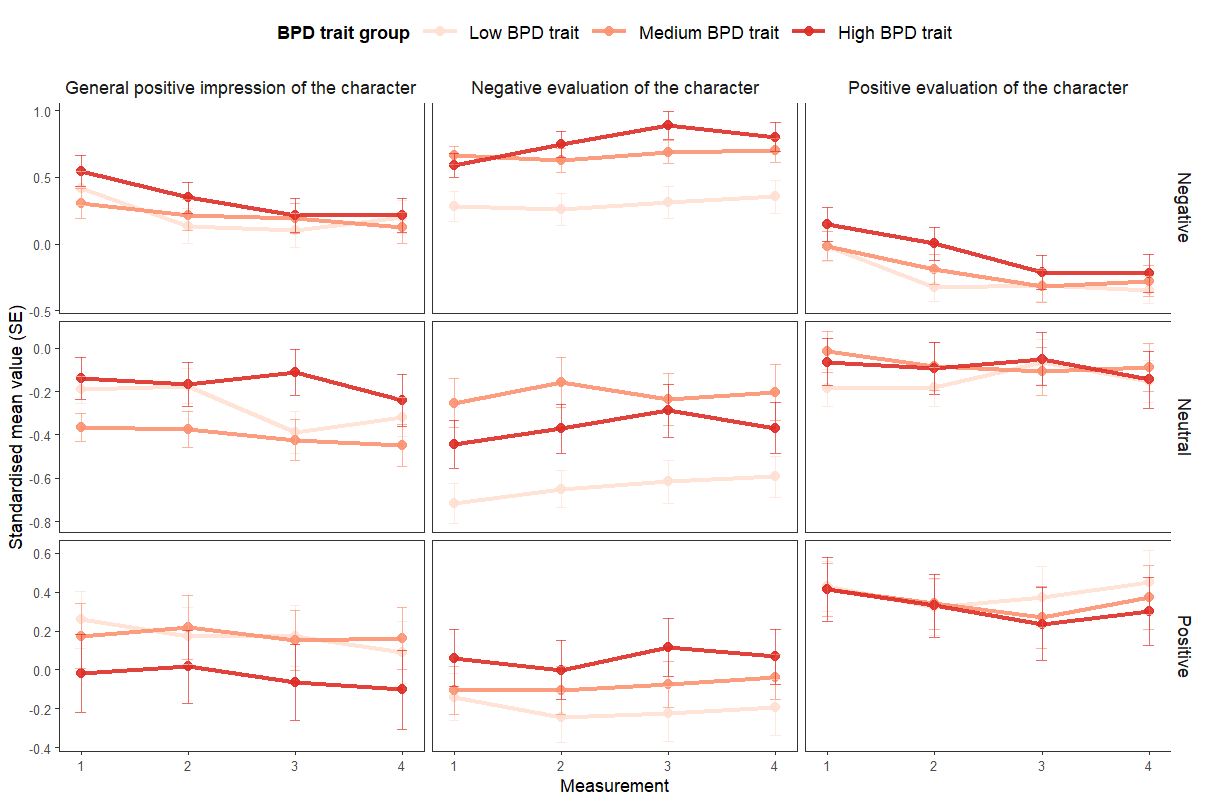


*Note*: The figure shows the character evaluation in columns and video valence as rows. The colors correspond to BPD trait groups that were created by cutting the distribution into three approximately similar sized groups (light red = low BPD, medium red = medium BPD and dark red = high BPD group). These groups were only used for visualization purposes and all statistical models used the continuous BPD values.
